# School-Age Children Are a Reservoir of Malaria Infection in Malawi

**DOI:** 10.1371/journal.pone.0134061

**Published:** 2015-07-24

**Authors:** Jenny A. Walldorf, Lauren M. Cohee, Jenna E. Coalson, Andy Bauleni, Kondwani Nkanaunena, Atupele Kapito-Tembo, Karl B. Seydel, Doreen Ali, Don Mathanga, Terrie E. Taylor, Clarissa Valim, Miriam K. Laufer

**Affiliations:** 1 Center for Malaria Research, Institute for Global Health, University of Maryland School of Medicine, Baltimore, Maryland, United States of America; 2 Department of Epidemiology, University of Michigan School of Public Health, Ann Arbor, Michigan, United States of America; 3 Blantyre Malaria Project, University of Malawi College of Medicine, Blantyre, Malawi; 4 Michigan State University, East Lansing, Michigan, United States of America; 5 National Malaria Control Program, Ministry of Health, Lilongwe, Malawi; 6 Department of Immunology and Infectious Diseases, Harvard School of Public Health, Boston, Massachusetts, United States of America; Université Pierre et Marie Curie, FRANCE

## Abstract

Malaria surveillance and interventions in endemic countries often target young children at highest risk of malaria morbidity and mortality. We aimed to determine whether school-age children and adults not captured in surveillance serve as a reservoir for malaria infection and may contribute to malaria transmission. Cross-sectional surveys were conducted in one rainy and one dry season in southern Malawi. Demographic and health information was collected for all household members. Blood samples were obtained for microscopic and PCR identification of *Plasmodium falciparum*. Among 5796 individuals aged greater than six months, PCR prevalence of malaria infection was 5%, 10%, and 20% in dry, and 9%, 15%, and 32% in rainy seasons in Blantyre, Thyolo, and Chikhwawa, respectively. Over 88% of those infected were asymptomatic. Participants aged 6–15 years were at higher risk of infection (OR=4.8; 95%CI, 4.0–5.8) and asymptomatic infection (OR=4.2; 95%CI, 2.7–6.6) than younger children in all settings. School-age children used bednets less frequently than other age groups. Compared to young children, school-age children were brought less often for treatment and more often to unreliable treatment sources. Conclusion: School-age children represent an underappreciated reservoir of malaria infection and have less exposure to antimalarial interventions. Malaria control and elimination strategies may need to expand to include this age group.

## Introduction

Despite widespread expansion of malaria control measures in the past decade, malaria remains a leading cause of morbidity and mortality, with children under five years of age and pregnant women most severely affected in Africa [1]. While standard interventions and surveillance policies have targeted these two groups at highest risk for malaria disease, older children and adults with *Plasmodium* infections, who are less often symptomatic, may play an important role in continued transmission. These groups have not traditionally been the focus of intensive detection and control strategies. To achieve malaria control and ultimately elimination, it may be important to identify and characterize these potential reservoirs of infection and transmission.

Older children and adults may serve as transmission reservoirs because acquired antimalarial immunity leads to persistent asymptomatic or minimally symptomatic infections that are less likely to be treated with antimalarial drugs than acute febrile illnesses that usually occur in young children [2]. Asymptomatic infections, representing a majority of all malaria infections [3], are generally low density, may be missed in surveillance strategies that rely on microscopy or antigen detection, and can persist between seasons and perpetuate transmission [4,5].

The epidemiological importance of older age groups may increase as elimination efforts intensify and transmission patterns change [6]. For example, in Kenya after nationwide net distribution, increased parasite prevalence was observed in asymptomatic school-age children [7]. Malaria treatment and control interventions in Zimbabwe resulted in a decrease in clinical malaria episodes, but the proportional reduction was greatest among children under five years of age, compared to older children and adults [8]. In Malawi, the most recent national Malaria Indicator Survey (MIS) demonstrated a decrease in microscopic prevalence from 43% to 28% between 2010 and 2012 among children under five years of age [9] following expanded community-level interventions in high-risk districts. However, within the under-five-year-old population, a subgroup analysis showed a significant sequential increase in prevalence from youngest to oldest subgroups, suggesting that prevalence may continue to increase in older children. Malaria infection prevalence data from all age groups are essential as control efforts focus on malaria elimination rather than disease control.

To better characterize infections, utilization of interventions, and health care seeking behaviors in all age groups, the Malawi International Center of Excellence for Malaria Research (ICEMR) has undertaken biannual community-based household surveillance in low and high transmission settings and seasons, using both microscopic and molecular malaria diagnostics. We hypothesized that older children not routinely included in malaria surveillance represent a reservoir of asymptomatic infections, and that this age-group has limited access to prevention and treatment interventions.

## Materials and Methods

### Surveys

Cross-sectional household surveys were conducted at the end of the dry season 2012 (September-October) and end of the rainy season 2013 (April-May), when malaria transmission is expected to be at its lowest and highest, respectively. Three districts in southern Malawi were chosen to represent the range of malaria transmission settings. Blantyre city is an urban highland setting with expected low transmission; Thyolo is a rural highland area with expected low to moderate transmission; and Chikhwawa is low-lying and rural with high transmission.

**Fig 1:**
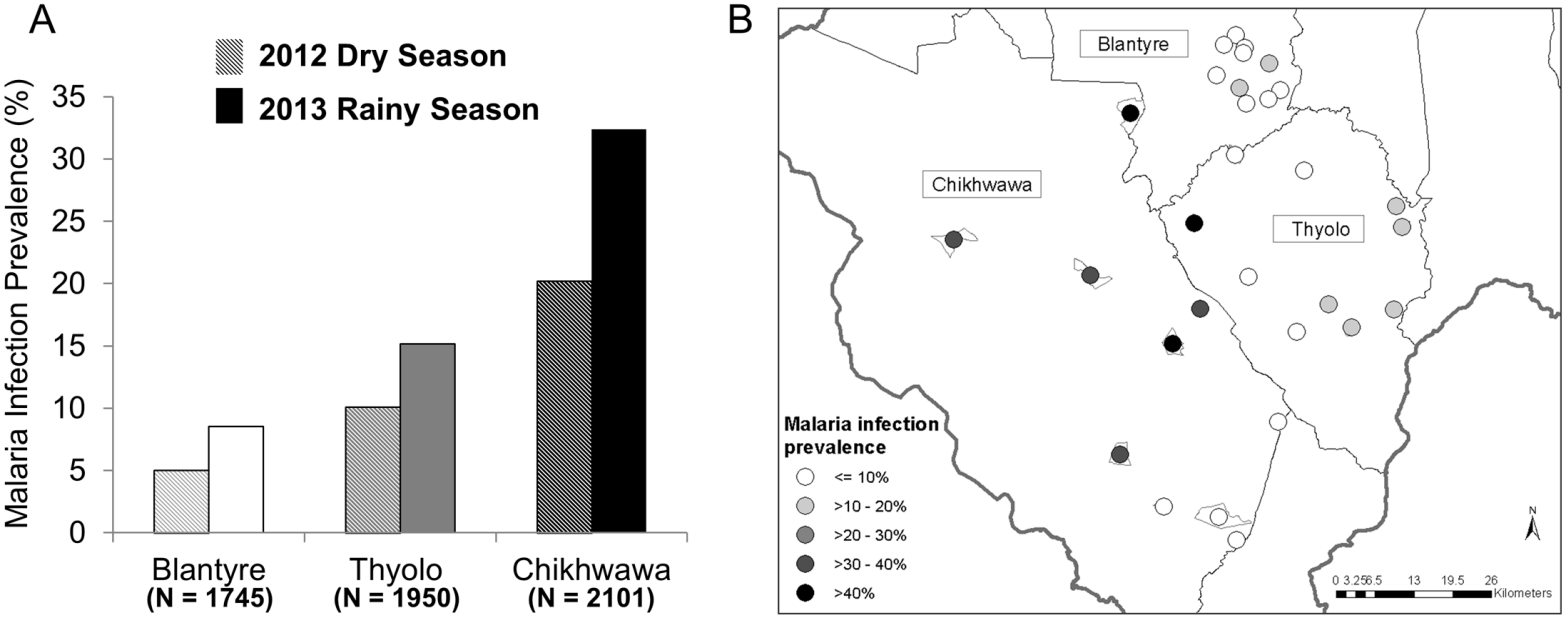
Malaria infection prevalence diagnosed PCR by season and district. Prevalence was heterogeneous across districts and seasons (A) and across sites within district (B). Pearson chi-square P values were <0.001 for comparisons of prevalence of parasitemia across districts in both seasons and were <0.005 for comparisons of prevalence between seasons. Likelihood ratio P values for homogeneity of prevalence within districts were < 0.001.

One compact segment containing 30 households in each of 10 enumeration areas (EAs) per district was selected using two-stage cluster sampling [10]. EAs were excluded if they met any of the following criteria: 1) EAs on the border between Chikhwawa and Thyolo; 2) Chikhwawa EAs >500m above sea level; 3) Thyolo EAs <500m above sea level. Three EAs were excluded based on these criteria and replaced with the next randomly selected EA. Selected districts and EAs are shown in Fig 1. The same selected households in each district were surveyed each season; all households within a given compact segment were visited on a single day. Households were excluded and replaced with another household within the compact segment if there were no adults over 18 years to provide consent.

Prior permission to survey each village was provided by village leaders. Written informed consent and assent were obtained for all adults and children, as appropriate. Questionnaires were administered in the local dialect. The study received ethical approval from University of Maryland Baltimore and Michigan State University Institutional Review Boards and University of Malawi College of Medicine Research and Ethics Committee.

### Study participants

Household members were defined as individuals who slept in the house for at least two weeks of the past month. Interview data about any household member (present or absent) was collected from a consenting adult household member; specimens were collected from those present at the time of survey.

### Data collection

Questionnaires were adapted from standardized MIS tools. Data were collected on android-based tablets using OpenDataKit (http://opendatakit.org) and managed using electronic data capture [11]. Data collected included household characteristics, socioeconomic variables, demographics, household net ownership and individual use, indoor residual spraying (IRS), recent fever, treatment seeking, source of treatment, and anti-malarial medication use within the previous two weeks. Age categories were: infants, < 6 months (intervention data only); young children, 6 months to 5 years; school-age, 6 to 15 years; and adults, ≥16 years. Individual net use was determined by asking if the individual slept under a net the previous night. Household characteristics included assessment of roof, wall, and floor materials; finished materials were defined as man-made (brick, cement, wood, etc) rather than natural. Medical treatment sources included: government health service, private health service, informal shop, and community health workers (CHWs). Axillary temperature was taken for all household members; blood samples were obtained from those ≥ 6 months of age for preparation of a thick smear for microscopy and dried blood spots on filter paper for polymerase chain reaction (PCR).

### Malaria diagnostic methods and infection definitions

For qualitative diagnosis of malaria infection, DNA was extracted from filter papers [12] and subsequently subjected to real-time PCR targeted to the *Plasmodium falciparum* lactate dehydrogenase gene [13]. Microscopy was conducted to distinguish microscopic from submicroscopic infections using published quality control methods [14].

Malaria infection was defined based on the PCR result. Asymptomatic malaria infection was defined as malaria infection without measured fever (temperature ≥37.5°C) or report of fever or rigors within the past 48 hours. Submicroscopic malaria infection was defined as malaria infection identified by PCR from individuals with malaria slides negative by microscopy. Review of at least 100 microscopic fields was required before a blood smear was determined negative for parasites.

### Statistical analysis

Malaria infection and intervention prevalence estimates by district, season and age group were compared using Pearson chi-square tests or Fisher’s exact test. Heterogeneity of prevalence across EA level within each district was assessed using logistic regression with likelihood-ratio tests. District comparisons for continuous variables were made using t-tests or Wilcoxon rank-sum tests, as appropriate. Individual counts per household and net were estimated using overdispersed Poisson regression.

Predictors of parasitemia and access to malaria interventions were identified using multivariate mixed models, with random intercept to account for hierarchical clustering by households and EA. Parasitemia outcomes modeled were: PCR-detected infection, asymptomatic infection among all infections, and submicroscopic infection among all infections. Intervention outcomes modeled were: net use among all individuals, treatment seeking among individuals with history of fever within two weeks, and source of treatment (government versus informal shop) among those with a history of seeking treatment. The primary predictor variable of interest was age group. To adjust for community-level malaria prevalence, we used data from the prior rainy season 2012 to create EA prevalence categories: low (<25^th^ percentile EAs); medium (25-75^th^ percentile EAs); or high (>75^th^ percentile EAs). District and season were forced into all models, and best subset regression was used to select final models based on p-value <0.05 or substantial change in the coefficients of variables included in models. Potential interactions were evaluated for each model. Since community IRS campaigns occurred in Chikhwawa district only after the 2012 rainy season, a Chikhwawa-specific model was fit to assess the effect of IRS on infection.

Data analysis was done with SAS version 9.3 (SAS Institute Inc., Cary, NC, USA).

## Results

### Population characteristics

During the two surveys, 1,833 households (911 dry season, 922 rainy season) and 7,653 individuals (3,889 dry season, 3,764 rainy season) were surveyed. Age distributions were similar across districts (Table 1). Household characteristics by district reflect lower socioeconomic status across several indicators (household materials, income source, education level) in the rural districts compared to Blantyre. Of 7,543 individuals over six months of age eligible to provide a blood sample, 5,796 provided samples. Of the 23% who did not, most were adult males who were not present. Among participants who did and did not provide samples, 31% and 28%, respectively, were school-age children.

**Table 1:** Household and individual characteristics by district.

	**Blantyre**	**Thyolo**	**Chikhwawa**
**Number of households surveyed**	(N = 601)	(N = 623)	(N = 609)
**Number of individuals per household, *mean ± SD***	4.3 *±* 1.9	3.9 *±* 1.7	4.3 *±* 1.8
**Highest level of education of household head or spouse, *n (%)***			
Never attended school	26 (4.4)	135 (21.7)	146 (24.0)
Primary education	232 (38.9)	367 (59.1)	373 (61.3)
Secondary education	316 (52.9)	110 (17.7)	86 (14.1)
University/college	23 (3.9)	9 (1.5)	4 (0.7)
**Electricity, *n (%)***	182 (30.4)	9 (1.5)	4 (0.7)
**Finished house materials** ^**a**^ **, *n (%)***	551 (92.1)	290 (46.7)	124 (20.4)
**Closed eaves *n (%)***	575 (96.0)	473 (76.2)	337 (55.4)
**Individuals surveyed**	(N = 2613)	(N = 2435)	(N = 2605)
**Female *n (%)***	1343 (51.8)	1367 (56.2)	1389 (53.6)
**Age category *n (%)***			
0–5 months	32 (1.2)	32 (1.3)	46 (1.8)
6 months–5 years	491 (19.0)	464 (19.1)	513 (19.9)
6–15 years	703 (27.2)	748 (30.8)	810 (31.4)
≥16 years	1358 (52.6)	1185 (48.8)	1214 (47.0)
**Women ≥12 years of age, *n (%)***	842 (32.2)	869 (35.7)	832 (31.9)
**Pregnant during survey** ^b^ **, *n (%)***	45 (5.3)	33 (3.8)	58 (7.0)

Abbreviation: SD = Standard Deviation

P values for chi-square tests comparisons across districts for household categorical variables were all <0.001. P values for comparisons of individual categorical variables were all <0.01 with the exception of pregnant during survey (P = 0.09). ANOVA was used for comparison across districts for the continuous variable household size where P<0.001. Dry season 2012 and rainy season 2013 are combined. Data analyzed for each season separately did not reveal relevant differences for the demographic and socioeconomic variables included in this table.

^a^Finished house materials defined as at least 2 of 3 house components (walls, floor, roof) finished versus natural or rudimentary

^b^By individual report among women ≥12 years of age

### Prevalence of malaria infection

Molecular results were obtained from 5,796 individuals. The prevalence of malaria infection in Blantyre, Thyolo, and Chikhwawa was 5%, 10%, and 20% in dry, and 9%, 15%, and 32% in rainy seasons. Rainy season prevalence was significantly higher than dry season prevalence in all districts (Fig 1A). Parasite prevalence varied significantly by EA within districts (P<0.001 in each district) (Fig 1B).

Overall, rainy season parasite prevalence was 14% among young children, 31% among school-age children, and 14% among adults (P<0.001). While multiple factors were associated with infection in bivariate analysis, multivariable analysis in all three districts, controlling for season and EA-level prevalence, revealed that school-age children had 4.8 (95% confidence interval [CI], 4.0–5.8) fold increased odds of infection compared to both younger children and adults (Table 2). Factors protective against infection included sleeping under a net the previous night and living in a house with finished materials. In Chikhwawa, the only district exposed to IRS, individuals from households sprayed within the last 12 months were also at decreased odds of infection (odds ratio [OR] = 0.4; 95%CI, 0.3–0.6) compared to those not sprayed, controlling for season and age.

**Table 2:** Risk factors for malaria infection.

	Bivariate			Multivariable		
	Unadjusted odds ratio	[95% CI]	P value	Adjusted odds ratio	[95% CI]	P value
**District**			<0.001			
Blantyre	-					
Thyolo	2.0	[1.5, 2.7]				
Chikhwawa	6.3	[4.8, 8.3]				
**EA prevalence**			<0.001			
≤ 25^th^ percentile (4%)	-			-		
25-75^th^ percentile (4–21%)	2.4	[1.8, 3.2]		2.3	[1.7, 3.2]	
> 75^th^ percentile (21%)	17.0	[12.7, 22.8]		20.7	[14.8, 29.0]	<0.001
**Rainy season**	1.7	[1.4, 2.2]	<0.001	2.0	[1.6, 2.5]	<0.001
**Female**	0.7	[0.6, 0.8]	<0.001			
**Age category**			<0.001			
6 months–5 years	-			-		
6–15 years	4.7	[3.9, 5.6]		4.8	[4.0, 5.8]	
≥16 years	1.0	[0.8, 1.2]		1.0	[0.8, 1.2]	<0.001
**Education level**			<0.001			
None	-					
Elementary	0.7	[0.5, 0.9]				
Secondary or college	0.3	[0.2, 0.4]				
**Finished house materials** ^a^	0.3	[0.3, 0.4]	<0.001	0.5	[0.4, 0.7]	<0.001
**Closed eaves**	0.4	[0.3, 0.5]	<0.001			
**Sought treatment within 2 weeks**	0.8	[0.6, 0.9]	0.01			
**Antimalarial med within 2 weeks**	0.8	[0.6, 1.0]	0.05			
**Slept under a net last night**	0.7	[0.6, 0.8]	<0.001	0.7	[0.6, 0.9]	<0.001
**Number of nets used by household**	1.0	[0.9, 1.1]	0.76			
**Community net use** ^b^			<0.001			
≤25^th^ percentile	-					
25-75^th^ percentile	1.6	[1.2, 2.1]				
>75^th^ percentile	3.2	[2.4, 4.4]				
**Number of individuals per household**	1.0	[0.98, 1.1]	0.17			
**Household net ownership (≥ 1 net)**	1.8	[1.3, 2.6]	<0.001			

Abbreviations: OR = Odds ratio; CI = Confidence interval; EA = enumeration area; IRS = insecticide residual spraying.

Mixed multivariate model represents the final model including district, season and additional variables with p-value <0.05 or that caused substantial change in the coefficients of other variables in the model. Wald type III P values and confidence limits are shown for each variable included in the model.

^a^Finished house materials defined as at least 2 of 3 house components (walls, floor, roof) man-made versus natural or rudimentary

^b^Each EA was assigned a community net use category based on the prevalence of net use by individuals within the EA as defined by quartiles.

### Prevalence of asymptomatic infections

**Fig 2:**
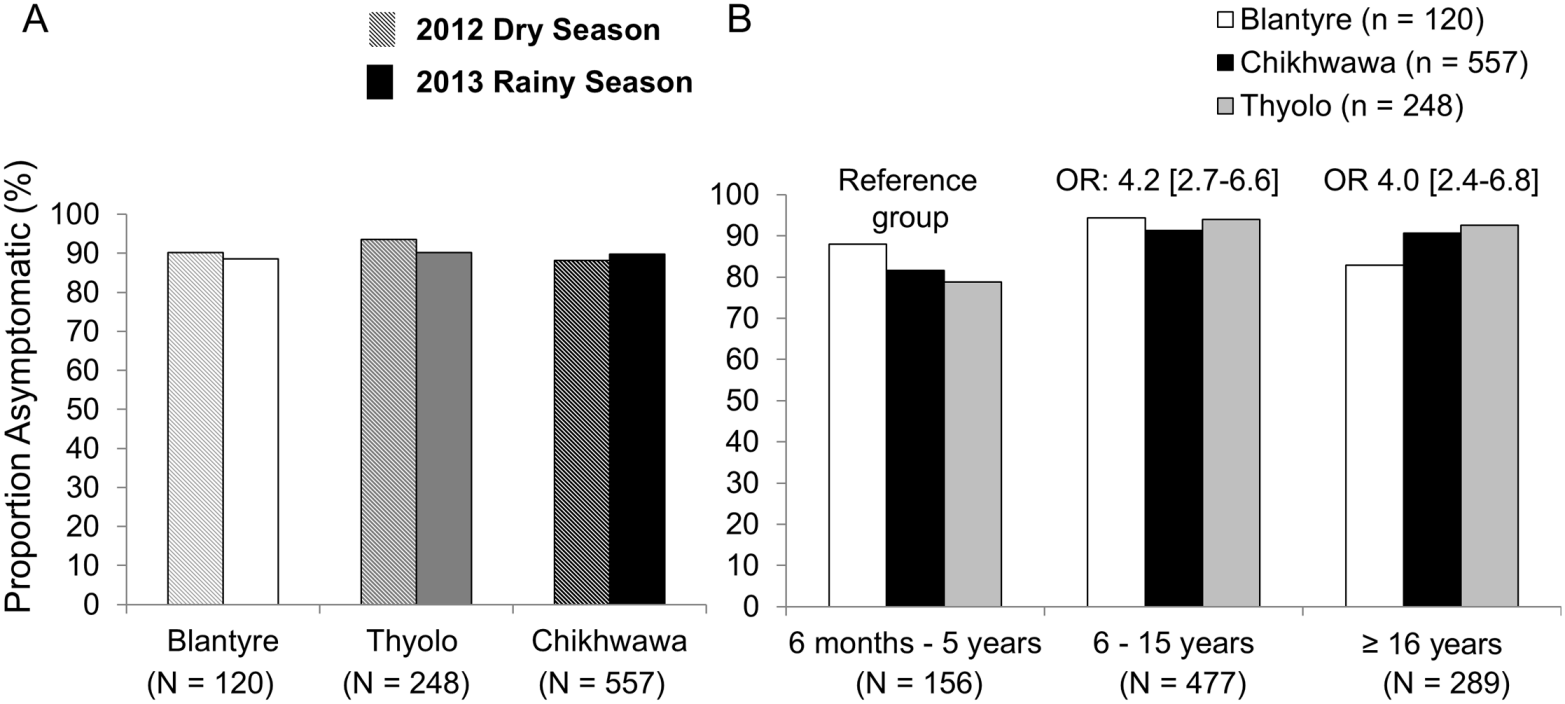
Asymptomatic infections among malaria-infected individuals by season and district (A) and by age group (B). Asymptomatic malaria was defined as PCR positive, with temperature <37.5°C and without reported fever within 48 hours. Prevalence differences were comparable (and Pearson chi-square P values >0.05) across districts in dry and rainy seasons and between seasons. In 2B, the odds of asymptomatic infection by age group were adjusted for season and sex and were statistically significant in Chikhwawa and Thyolo but not in Blantyre (P_interaction age and district_ = 0.001).

Almost all infected individuals were asymptomatic; the proportion of asymptomatic infections among infected individuals was similar across seasons (P = 0.88) and districts (P = 0.59) (Fig 2A). Among malaria-infected participants, school-age children and adults had a four-fold increased odds of asymptomatic infection compared to younger children respectively after adjustment for potential confounders (Fig 2B). These differences in odds by age group varied by district (P_age-district-interaction_ = 0.001), and were not observed in Blantyre. Males had twice the odds of asymptomatic infection as females (OR = 1.9; 95%CI, 1.4–2.8). The association of age and likelihood of asymptomatic infection among those infected was not affected by other socioeconomic variables or net use.

### Prevalence of submicroscopic infections

**Fig 3:**
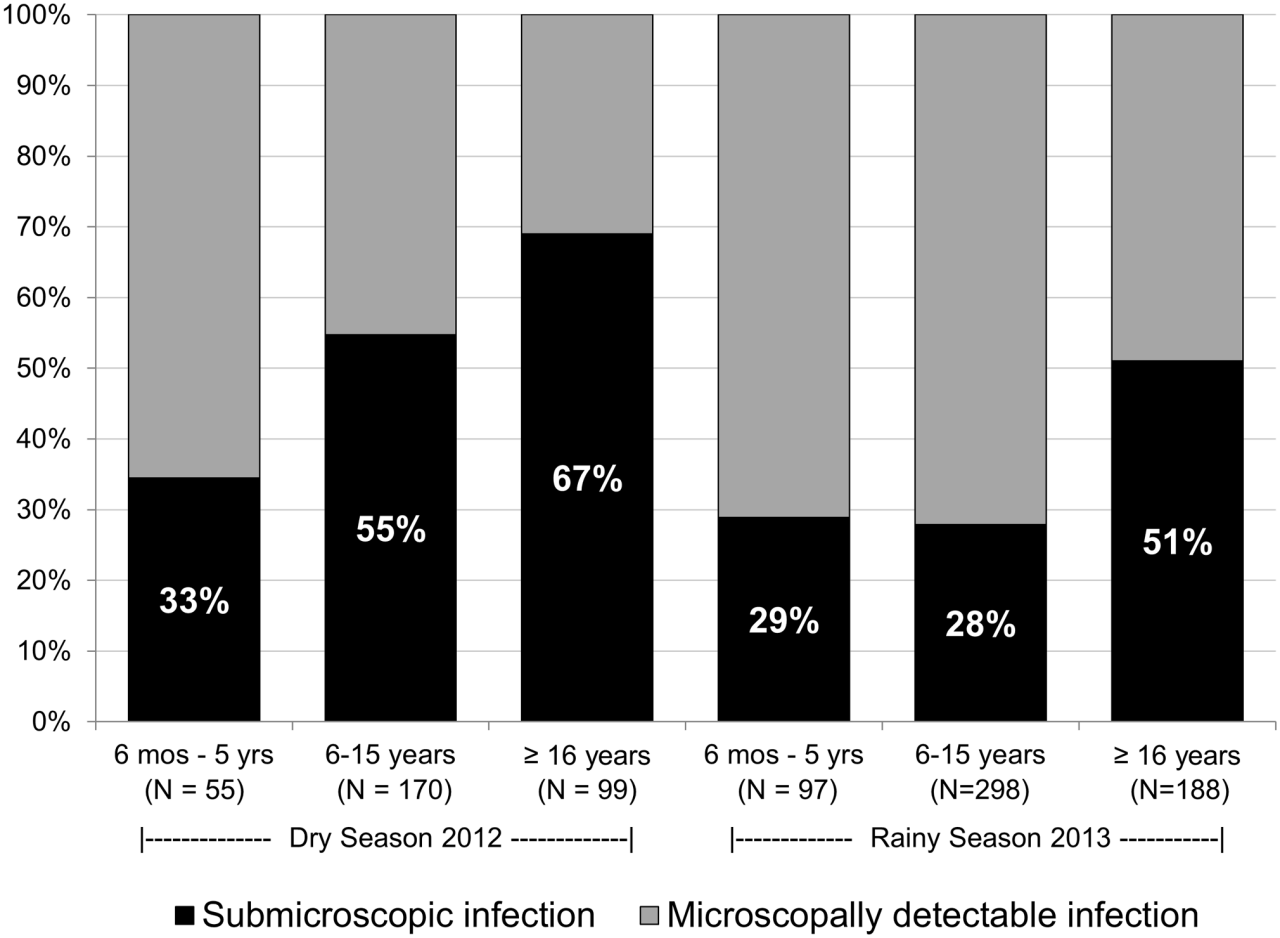
Proportion with submicroscopic infection among all malaria infections by age group. Association of submicroscopic parasitemia with age and season was unchanged after adjustment for district, gender, net use, net ownership, and house materials.

In both crude and adjusted analyses, submicroscopic infections represented the majority of infections in the dry (55%), but not in the rainy season (36%, P<0.001). In the rainy season, the proportions of submicroscopic infections were comparable between school-aged and younger children, but in the dry season, school-age children were more likely to have submicroscopic infection than younger children (55% vs 33%, P = 0.005). In adults, most infections were submicroscopic in both dry and rainy seasons (Fig 3). Submicroscopic infections were also more likely in the low (OR = 2.1; 95%CI, 1.2–3.8) and middle (OR = 2.0; 95%CI, 1.3–2.9) prevalence EAs versus the highest prevalence EAs.

### Access to interventions—bednets

The reported prevalence of sleeping under a net the previous night was 67%, 64%, and 76%, in the rainy season and 61%, 49%, and 63% in the dry season in Blantyre, Thyolo, and Chikhwawa, respectively. Prevalence of household net ownership and individual net use during the rainy season are shown by district in Table 3. Analyses of dry season data revealed comparable results. There were significant differences in net use based on age: during the rainy season, school-age children were less likely to have slept under a net (57%) compared to young children (76%, P<0.001) and adults (74%, P<0.001; Table 3).

**Table 3:** Use of malaria control interventions by district and age group in the rainy season.

**Household interventions**	Blantyre	Thyolo	Chikhwawa					
	(N = 303)	(N = 313)	(N = 306)					
**Household owns ≥1 net, *n (%)***	255 (84.2)	267 (85.3)	285 (93.1)	***				
**Individuals per net used per HH, *mean ± SD***	2.3 ± 0.4	2.2 ± 0.4	2.5 ± 0.4	***				
**Indoor residual spraying within past 12 months, *n (%)***	5 (1.7)	0	223 (73.1)	***				
**Individual interventions**	Blantyre	Thyolo	Chikhwawa		≤ 5 years	6–15 years	≥16 years	
	(N = 1281)	(N = 1196)	(N = 1287)		(N = 790)	(N = 1130)	(N = 1831)	
**Individual slept under a net last night, *n (%)***	862 (67.3)	763 (63.8)	975 (75.8)	***	600 (76.0)	645 (57.1)	1345 (73.5)	***
**Fever reported within 2 weeks** ^a^ **, *n (%)***	227 (17.7)	247 (20.7)	342 (26.6)	***	222 (28.1)	180 (15.9)	413 (22.6)	***
**Sought treatment or medical advice in past 2 weeks, *n (%)***	188 (14.7)	192 (16.1)	283 (22.1)	***	189 (23.9)	142 (12.6)	332 (18.3)	***
**Sought treatment <48 hours from first fever** ^b^	87 (58.8)	80 (49.7)	106 (42.7)	**	96 (59.3)	58 (46.4)	119 (44.1)	**
**Treatment source, *n (%)*** ^b^								
Government health service	85 (55.2)	83 (50.9)	142 (56.6)	NS	110 (66.7)	57 (45.2)	143 (51.6)	***
Shop	50 (32.5)	66 (40.5)	100 (39.8)	NS	41 (24.9)	60 (47.6)	119 (43.0)	***
Private health service	18 (11.7)	5 (3.1)	7 (2.8)	***	6 (3.6)	7 (5.6)	17 (6.1)	NS
Community health worker	1 (0.7)	9 (5.5)	2 (0.8)	***	11 (6.7)	3 (2.4)	0	***

Abbreviations: HH = Households; SD = Standard Deviation; NS = non-significant;

* P<0.05;

** P<0.01;

*** P<0.001.

P values were calculated using Pearson chi-square tests or Fisher exact tests were used for comparisons across districts and across age groups for categorical variables as appropriate. ANOVA was used for comparison across districts for the continuous variable.

^a^ Fever defined as reported fever or chills/rigors within 2 weeks or measured fever at the time of survey.

^b^ Among those with fever within 2 weeks and seeking treatment (n = 568).

### Access to interventions—treatment

Overall, 22% of individuals had a history of fever within the previous two weeks; of these 18% reported seeking treatment (Table 3). Across all districts, the most common treatment source was government health services (53%), followed by informal shops (41%) and other sources of treatment (private providers and CHWs).

There were significant differences in malaria treatment seeking by age: school-age children were brought for any treatment less frequently (P<0.001) and were less likely to be brought for treatment within 48 hours of onset of fever than younger children (P = 0.008, Table 3). These results were not affected by adjusting for socioeconomic factors, yet households where the head had at least secondary or university education were more likely to bring a child of any age for treatment than households with less education (OR = 1.6; 95%CI, 1.1–2.4). Among those with fever seeking treatment, in both crude and adjusted (Table 4) analyses, younger children were more commonly taken to a government health facility while school-age children were more commonly taken to an informal shop.

**Table 4:** Predictors for seeking treatment outside of the formal health sector (e.g. a local shop) versus at a government health center.

	Bivariate			Multivariable^a^		
	Odd ratio	[95% CI]	P value	Odds ratio	[95% CI]	P value
**District**						
Blantyre	-			-		
Thyolo	1.3	[0.9, 2.0]		1.0	[0.6, 1.7]	
Chikhwawa	1.4	[0.9, 2.1]	0.3	0.9	[0.5, 1.6]	0.9
**Rainy season**	0.7	[0.5, 0.99]	0.04	0.7	[0.5, 0.98]	0.04
**Female**	1.0	[0.8, 1.3]	0.9			
**Age category**						
6 months–5 years	-			-		
6–15 years	2.2	[1.5, 3.4]		2.6	[1.7, 3.9]	
≥16 years	1.9	[1.4, 2.6]	<0.001	2.3	[1.6, 3.2]	<0.001
**Sought treatment <48 hours from first fever**	2.2	[1.6, 3.0]	<0.001	2.5	[1.8, 3.4]	<0.001
**Education level**						
None	-					
Elementary	1.1	[0.7, 1.8]				
Secondary or college	1.0	[0.6, 1.7]	0.8			
**Finished house materials** ^b^	0.6	[0.4, 0.9]	0.004	0.5	[0.3, 0.8]	0.005

^a^Mixed multivariate model represents the final model including district, season and additional variables with p-value <0.05 or that caused substantial change in the coefficients of other variables in the model. Wald type III P values and confidence limits are shown for each variable included in the model.

^b^Finished house materials defined as at least 2 of 3 house components (walls, floor, roof) man-made versus natural or rudimentary

## Discussion

In this large cross-sectional study, school-age children had the highest prevalence of malaria infection and were not likely to report associated symptoms. School-age children were also more likely to be submicroscopic carriers in the dry season, and to have lower use of both nets and treatment. These findings were consistent across settings and seasons despite significantly different infection prevalence by setting and season.

This is the first study to link community prevalence of malaria among school-age children to decreased access to malaria interventions. The results are consistent with results from more limited prior studies [8,15–17], but none have reported prevention or treatment-seeking behavior in the school-age group.

In Malawi, as in other endemic regions, school-age children sleep under nets less often than any other age group. A study examining national survey data from 18 African malaria-endemic countries identified net use patterns similar to those seen in our study: mothers slept under nets with their young children, while other household members slept under nets less frequently [18]. Women of childbearing age in antenatal and vaccination clinics have been the focus of targeted education and net distribution activities, and this focus may help to explain the lower bednet use observed in school-age children. The vulnerability of young children to malaria morbidity and mortality is often well-known in malaria-endemic communities. Older children and adults are assumed to be less at risk and may use bednets less consistently. The lack of utilization or access to prevention methods such as insecticide-treated nets may, in part, explain elevated infection prevalence among school-age children. These infections are more often asymptomatic and thus less likely to be treated, leading to the persistence of reservoirs of potential transmission.

The semi-immune status of older children and adults spares them from the clinical symptoms that commonly prompt care-seeking. The fact that older children often appear less sick may also lead caregivers to seek care from a nearby shop rather than traveling to a formal government or private health center. Despite free access to government health services in Malawi, we found that local shops were used by nearly half of the population with fever, and shops were more likely used when treatment was sought early. Local shops are a common source of care for febrile individuals in endemic countries [19,20], and shopkeepers may provide inappropriate medications for customers with symptoms of malaria [21,22].

Beyond lack of access to prevention and treatment, entomologic factors such as predominance of outdoor biting vectors may be related to the observed elevated malaria prevalence in school-age children and adults [7]. Older children and adults typically spend more time outdoors during field work and may remain outside the house later than young children.

Logistical constraints may have introduced non-response and recall biases to our results, but should not have impacted the validity of our main conclusions. Because our cross-sectional survey teams worked during the day, school-age children and adults at work were more likely to be absent than younger children and mothers. The survey team made particular efforts to capture household members by alerting communities of the planned survey date in advance and waiting at the site for school-age children and working adults who returned at the end of the day. The proportion of school-age children who did and did not provide a blood specimen was similar. Adult men appear to have been consistently under-represented; if they were at higher risk of malaria infection, we may have underestimated the prevalence of malaria infection in this age group. Recall bias may have resulted in an underestimate of the proportion of asymptomatic malaria in younger children. A household head was asked about use of bednets, recent symptoms and treatment seeking for all household members; therefore, symptoms may have been identified more readily in young children, who are more likely to be at home with a parent all of the time. The definition of asymptomatic malaria based on lack of fever and rigors is specific. Exclusion of additional malaria-related symptoms would have made the definition of asymptomatic more sensitive; however, detailed symptom history was not available. Many investigators have noted the complexities of defining asymptomatic malaria infection, especially in high transmission settings [4,23]. In this community-based survey, comparisons of proportion asymptomatic using a slightly more sensitive definition are unlikely to have led to different conclusions. These possible biases would not have substantially affected our estimates of inverse association between access to interventions and parasite prevalence in school-age children.

This study highlights the importance of including school-age children and adults in malaria surveillance activities, given their contribution to the pool of asymptomatic reservoirs of infection and potentially of transmission. Affordable and sensitive diagnostic methods are needed to detect low levels of infection. In addition, control measures directed toward high-transmission areas defined by political boundaries rather than environmental or socioeconomic risk factors may not capture hot spots for malaria infection. Appropriate surveillance and intervention strategies should be implemented to identify all infected individuals and capture the heterogeneity of malaria transmission within close geographic proximity.

There is urgent need to target school-age children for malaria prevention efforts. Educational efforts may be highly effective in promoting bednet use and improving health-seeking behavior in this high prevalence population to maximize utilization of existing resources. With high prevalence of submicroscopic infection identified in this study and lack of a point-of-care test to reliably detect low density infections, mass drug administration programs may prove more useful than screening and treatment approaches for a school- or community-based intervention targeting all infected individuals.

## Conclusions

School-age children are a key reservoir for malaria infection and this population should be included in surveillance and control activities in all transmission settings and seasons in malaria-endemic countries. Prevention and treatment programs are utilized less commonly in school-age compared to younger children; novel interventions and strategies to target this group are needed.
